# Differential sensitivities of bladder cancer cell lines to resveratol are unrelated to its metabolic profile

**DOI:** 10.18632/oncotarget.15041

**Published:** 2017-02-02

**Authors:** Yang Yang, Chuangang Li, Hong Li, Moli Wu, Changle Ren, Yuhong Zhen, Xiaochi Ma, Yunpeng Diao, Xiaodong Ma, Sa Deng, Jia Liu, Xiaohong Shu

**Affiliations:** ^1^ College of Pharmacy, Dalian Medical University, Dalian, Liaoning, China; ^2^ Academy of Integrative Medicine, Dalian Medical University, Dalian, Liaoning, China; ^3^ Surgery Department of The Second Affiliated Hospital, Dalian Medical University, Dalian, Liaoning, China; ^4^ College of Basic Medical Sciences, Dalian Medical University, Dalian, Liaoning, China; ^5^ Surgery Department of Dalian Municipal Central Hospital, Dalian Medical University, Dalian, Liaoning, China

**Keywords:** bladder cancer, resveratrol, metabolism, chemosensitivity, sulfotransferase 1A1

## Abstract

Resveratrol (RV) is a natural polyphenol compound with a wide range of activities, including inhibition of human bladder cancer (HBC) cell growth. Because RV is rapidly metabolized and has poor bioavailability, it is unclear whether the antitumor activity is due to RV or its metabolites. We therefore used liquid chromatography-mass spectroscopy, qRT-PCR, immunocytochemistry and western blotting to evaluate the metabolic profile and biotransformation of RV in the T24 and EJ HBC cell lines. Both T24 and EJ cells generated the same RV metabolite, RV monosulfate (RVS), and both exhibited upregulation of the RV-associated metabolic enzyme SULT1A1 (sulfotransferase). Despite these similarities, T24 cells were more sensitive to RV than EJ cells, yet T24 cells exhibited no sensitivity to an RVS mixture (84.13% RVS). Primary rat bladder epithelial cells showed no adverse effects when exposed to a therapeutic dose (100 μM) of RV. The differences in RV sensitivity between the two HBC cell lines did not reflect differences in the RV metabolic profile or SULT1A1 expression. Because RV exhibited stronger antitumor activity and better safety than RVS, we conclude that RV has significant therapeutic potential for HBC treatment, provided individual differences are considered during clinical research and application.

## INTRODUCTION

The combination of surgical operation with radiotherapy and adjuvant intravesical administration is the conventional treatment strategy for human bladder cancer (HBC) [1]. The ideal intravesical agent should be sensitive to HBC cells, and could be absorbed easily in the carcinoma cells but exert fewer side effects on normal tissue cells. Resveratrol (3,5,4’-trihydroxy-*trans*-stilbene, RV, Supplementary Figure 1A), a natural dietary polyphenol, possesses anti-cancer and other beneficial pharmacological activities [2–4]. Furthermore, the lipophilic characteristic of RV caused by its basic structural skeleton of the central carbon-carbon double bond conjugated with two benzene rings (Supplementary Figure 1A), which would lead it more easily to be absorbed by the bladder mucosa cells, and thus could reach the effective drug concentration in the carcinoma cells and exert better biological activities [5, 6]. With the characteristics of adjuvant intravesical therapy for bladder cancer, RV may be a viable candidate, especially in bladder cancer perfusion chemotherapy.

Since Jang *et al*. demonstrated that RV possessed cancer chemopreventive activity [3], studies on the bioactivity of RV have increased rapidly [7–9], and RV's anti-tumor effect represents some of the most convincing and intriguing [7–10]. It was reported that RV could restore PTEN expression by targeting oncomiRs of the miR-17 family in prostate cancer [11], and also could inhibit STAT3 activation, enhancing autophagy and apoptosis in rat orthotopic glioblastoma [12]. Short-term exposure RV could cause growth inhibition and apoptosis of HBC EJ cells *in vitro* and *in vivo* [13]. And as we all know, RV could be metabolized rapidly and produce various metabolites such as RV glucuronide or/and RV sulfate conjugates (Supplementary Figure 1) [14–18]. It was found that RV could be metabolized to RV sulfates in human breast cancer MB-MDA-231 and ZR-75-1 cells [14], human medulloblastoma UW228-3 [17], human glioblastoma LN-18 and U251 cells [19, 20]. However, RV glucuronide was found as the main metabolite in rat glioblastoma RG2 and C6 cells, and showed discrepant metabolic patterns between human and rat glioblastoma cells [20]. So far, little work has been carried out to explore the metabolism of RV in HBC EJ and T24 cells. Thus, how RV exerts its bioactivity in bladder cancer becomes an interesting issue, either by RV parent compound or its metabolites, or both RV and its metabolites synergistically exert the beneficial effect? To clarify this ambiguity, we analyzed RV's metabolic pattern in HBC T24 and EJ cells, then biotransformed its major metabolite *in vitro* and tested its bioactivity to ascertain the effective bioactive form of RV, and further checked the safety of the active compound at the therapeutic dosage to evaluate RV's clinic medicinal value.

## RESULTS

### Responses of BC cells to RV

**Figure 1:**
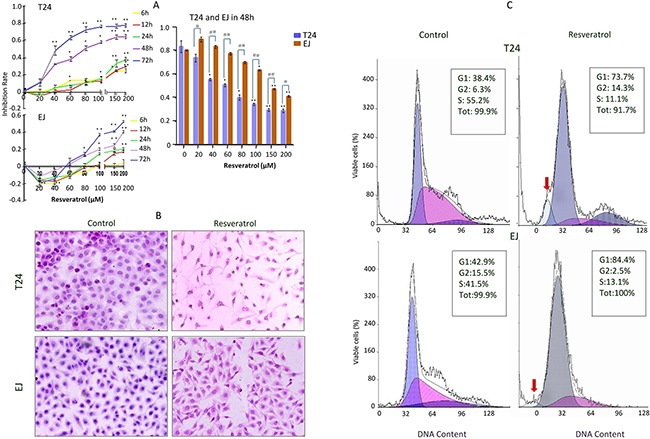
Chemosensitivity evaluation of resveratrol to T24 and EJ cells **A**. Effect of resveratrol treatment on human bladder cancer (HBC) T24 and EJ cells. Cells were incubated with different concentrations (0, 20, 40, 60, 80, 100, 150 and 200μM) resveratrol for different time periods (0, 6, 12, 24, 48 and 72h), respectively, and then the cells number was determined by MTT as described in the Materials and Methods. Data are presented as means ± S.D. of three independent experiments. Bars means standard errors, *P<0.05, **P<0.001 reveal significant difference between RV-treatment and Control HBC cells. ^#^P<0.05, ^##^P<0.001 show significant different between T24 RV-treatment cells and EJ RV-treatment cells. **B**. HE morphological staining performed on T24 and EJ cells without (Control) and with 100μM RV (Resveratrol) incubation for 48 hours (100×). Cells at a density of 4×10^5^ cells per well were placed in dishes with coverslips, then T24 and EJ cells were treated without (Control) and with (Resveratrol) 100μM resveratrol treatment for 48h. Cells coverslips were harvested for examination and T24 cells exhibited more obviously spindle-shaped change than EJ cells. **C**. Flow cytometry analysis on the fractionation of cell cycles and apoptotic cells in T24 and EJ cell populations without (Control) and with (Resveratrol) 100μM resveratrol incubation for 48 hours. Red arrows, indicate the peak of apoptotic cells. Data revealed a presentative experiment in triplicate with similar results.

To explore the biological activity and the effective dosage of RV in HBC T24 and EJ cells, MTT assay was carried out. As shown in Figure 1A (left), after incubation with 100μM RV for 6h, 12h, 24h, 48h and 72h, the inhibition ratio of T24 cells was 15.3±0.3 %, 13.6±0.3 %, 16.5±1.8 %, 58.5±1.5 % and 76.6±1.6 %, respectively. While the inhibition ratio of EJ cells was 2.4±0.3 %, 2.5±0.2 %, 15.1±1.1 %, 20.1±1.5 % and 37.3±1.6 % after incubation with 100μM RV for 6h, 12h, 24h, 48h and 72h, respectively. The above results showed that RV could induce a significant time-dependent growth inhibition to T24 cells, but the proliferation of EJ cells was less suppressed (Figure 1A) [21]. Meanwhile, Figure 1A (right) also presented a concentration-dependent inhibition in T24 and EJ cells after incubation with 0, 20μM, 40μM, 60μM, 80μM, 100μM, 150μM and 200μM RV, respectively.

The RV-sensitivity of HBC cells was further evaluated by hematoxylin and eosin (HE) staining, as shown in Figure 1B, we found the majority of T24 cells presented spindle-shaped, segments of cell bodies, and detached from the culture plate after exposure to 100μM RV for 48h. But compared with T24 cells, there was no obvious morphologic change in EJ cells. And the 100μM-RV 48h-treatment was used for the further experiments. Flow cytometry (FCM) analyses showed that the G1 and S fractions were 38.4% and 55.2% in normally cultured T24 cells, but changed to 73.7% and 11.1% after 100μM RV treatment (Figure 1C). The percentages of G1 and S phase of EJ cells were 42.9% and 41.5% under normal culture condition and become 84.4% and 13.1% after 100μM-RV treatment for 48h. The above results indicated RV could induce G1 phase cell cycle arrest in HBC cells (Figure 1C).

### RV monosulfate (RVS) was the major metabolite in HBC cells

**Figure 2:**
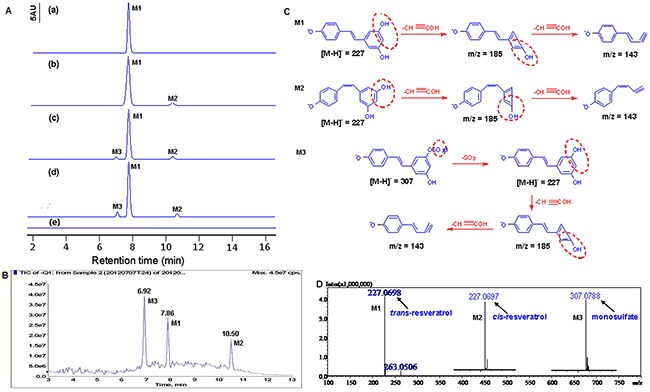
Identification of resveratrol's metabolites in HBC T24 cells **A**. HPLC chromatography analysis. (a) *trans-*resveratrol standard was dissolved in methanol and analyzed by HPLC (M1, *t_R_*=7.86); (b) The culture media incubation with resveratrol without T24 cells for 48h (M1, *t_R_*=7.86; M2, *t_R_*=7.02); (c) The T24 cells lysate was analyzed after incubation with 100μM resveratrol for 48h (M1, *t_R_*=7.89; M2, *t_R_*=7.06; M3, *t_R_*=10.38); (d) The supernatant of T24 cells was analyzed after incubation with 100μM resveratrol for 48h (M1, *t_R_*=7.87; M2, *t_R_*=7.01; M3, *t_R_*=10.41). (e) The T24 cells treated without resveratrol as Control. **B**. MS analyses of resveratrol metabolites in T24 cells. Total ion chromatogram (TIC) of the supernatant of T24 cells treated with 100μM resveratrol for 48h. Peak M1, M2 and M3 indicated retention time corresponding to different mass composition of metabolites; **C**. Proposed mechanism for the decomposition of the m/z 227 [M-H]^-^ ion of resveratrol, the decomposition of the m/z 227 [M-H]^-^ and m/z 307 [M-H]^-^ ion of metabolites. **D**. Shimadzu LC-MS-IT-TOF-based HRMS analysis of resveratrol metabolites in T24 cells. Arrows labeled as M1, M2 and M3 indicated the exact [M-H]^-^ molecular ion weight of 227.0698 (C_14_H_11_O_3_, calculated m/z 227.0708), 227.0697 (C_14_H_11_O_3_, calculated m/z 227.0708), 307.0788 (C_14_H_11_SO_6_, calculated m/z 307.0276), respectively. In Figure 3, M1 represents *trans*-resveratrol, M2 represents *cis*-resveratrol and M3 represents resveratrol monosulfate (RVS), respectively.

To identify the RV's metabolite(s) in HBC T24 and EJ cells, the cells and the conditioned media were collected after 100μM RV incubation for 48h, then were purified with solid phase extraction (SPE) to eliminate the interferer, and subsequently were analyzed by high performance liquid chromatography (HPLC), liquid chromatography-mass spectrometry (LC-MS) and high-resolution mass spectrometry (HRMS). For the metabolic profile of the T24 cells is similar to EJ cells (Supplementary Table 1, Supplementary Figure 2), we only showed the identification of T24 cells and its culture media (Figure 2). The T24 cell-free media which only contained RV was incubated for 48h as the background control. HPLC analyses showed that only one compound, *trans*-RV, was found in the standard group (Figure 2A(a), M1); two compounds, *trans*-RV and *cis*-RV, were presented in the control group (Figure 2A(b), M1, M2); and three major compounds could be detected both in T24 cell lysates (Figure 2A(c), M1, M2, M3) and condition media of T24 cells (Figure 2A(d), M1, M2, M3), which were later proved to be *trans*-RV, *cis*-RV and RVS according to their retention time and molecular weight. Compared with the above data, the T24 cells treated without RV showed no compound peak as control (Figure 2A(e)).

The above three major molecules were identified by LC-MS/MS using a combination of full and selected ion scanning techniques. Total ion chromatogram (TIC) of the T24 cells treated with 100μM RV for 48h were listed in Figure 2B, the peaks of M1, M2 and M3 could be detected from the chromatograms that represent *trans*-RV (M1), *cis*-RV (M2) and RVS (M3), respectively.

For further identify the RV metabolite(s) in HBC T24 and EJ cells, a combination of full and selected ion scanning of MS coupled with LC techniques was used. As illustrated in Figure 2C, the [M-H]^–^ spectrum of M1 characterized by its molecular ion at m/z 227 which generated a series of fragment ions at 185 and 143. The fragment ion at m/z 185 was generated from m/z 227 after loss of 42amu (C_2_H_2_O), and the m/z 185 was further fragmented to m/z 143 after the loss of 42amu (C_2_H_2_O), which corresponded to *trans*-RV (Figure 2C, M1). Another metabolite (M2) was considered as an isomeric RV with mass spectral features identical to M1, and the fragment ions showed m/z at 185 and 143 attributed to *cis*-RV (Figure 2C, M2). The [M-H]^-^ ion of M3 showed the dissociation molecule ions of m/z 307 and 227, respectively, the ion corresponding to RV (m/z 227) after losing 80amu, a sulfate moiety, from the RVS, then the m/z 227 was fragmented to m/z 185 for the further loss of 42amu (C_2_H_2_O) from RV, which appeared to be the main characteristic fragmentation pathway of RVS (Figure 2C, M3), and was also reported somewhere else [17, 22].

HRMS was applied to further confirm the RV metabolite(s), which showed the [M-H]^–^ molecular ion exact mass as 227.0698 (C_14_H_11_O_3_, calculated m/z 227.0708), 227.0697 (C_14_H_11_O_3_, calculated m/z 227.0708) and 307.0788 (C_14_H_11_SO_6_, calculated m/z 307.0276), which was consistent with the report of LC-MS/MS and corresponded to *trans*-RV, *cis*-RV and RVS, respectively (Figure 2D) [17].

### RV metabolic process in HBC cells

**Figure 3:**
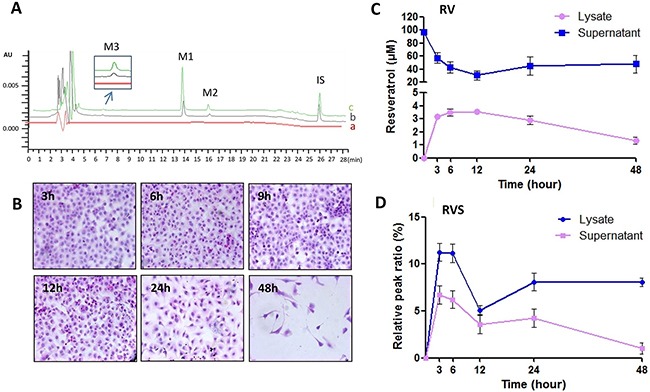
RV metabolic pattern in HBC T24 cells **A**. Representative HPLC/DAD analysis of resveratrol in T24 cells. (a) HBC T24 cells treated without RV as Control; (b, c) T24 cells treated with 100μM resveratrol for 48h, cell supernatant (b) and cell lysates (c) spiked with 1,8-dihydroxy anthraquinone (internal standard, IS). Peaks: M1. *trans*-resveratrol, *t_R_*=13.82min; M2. *cis*-resveratrol, *t_R_*=15.97min; M3. resveratrol monosulfate (RVS), *t_R_*=6.67min; IS. 1,8-dihydroxy anthraquinone, *t_R_*=25.93min (Internal standard/IS). **B**. Morphologic changes were evaluated by HE staining (100×), and T24 cells showed neither observable growth arrest nor morphological change until 24h resveratrol incubation. **C** & **D**. Quantification of RV and RVS in T24 cells. Resveratrol concentrations in the cell lysates and supernatant after 100μM resveratrol treatment for 3, 6, 12, 24 and 48h, respectively.

To evaluate the correlation between RV metabolism and its pharmaceutical activity, the RV metabolites in T24 and EJ cells and their supernatant were analyzed by HPLC, and the cell morphology was evaluated by HE staining (Figure 3). The supernatant and lysate of T24 cells were collected for HPLC analysis after 100μM RV treatment for 3h, 6h, 9h, 12h, 24h and 48h, respectively (Figure 3A). The HPLC results showed that RVS peak was observed as early as 3h after drug treatment (Figure 3C, 3D), but the T24 cells showed neither growth arrest nor morphological change until 24h-100μM RV treatment (Figure 3B), and the RVS didn't dominate in the supernatant and lysate (Figure 3C, 3D), although T24 cells showed distinctly growth inhibition in 48h-RV treatment (Figure 3B), which suggested that the RV metabolism was preceded to growth inhibition in T24 cells. Meanwhile, EJ cells showed a similar RV metabolic pattern with T24 cells. However, compared with T24 cells, EJ cells still did show neither obvious growth arrest nor morphological change till 48h RV-treatment (Supplementary Figure 2).

### RV upregulated SULT1A1 expression

**Figure 4:**
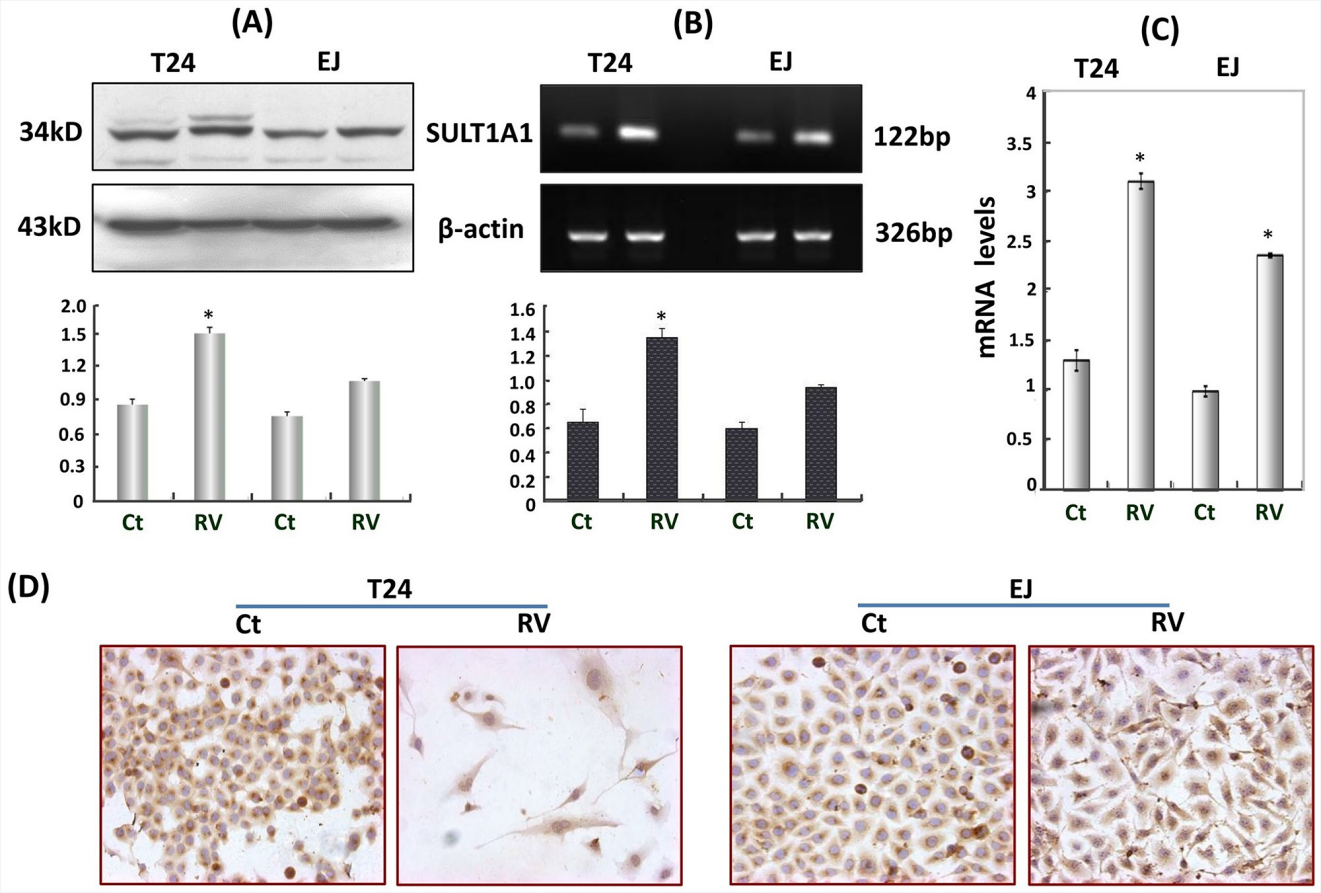
Resveratrol upregulated SULT1A1 expression in T24 and EJ cells **A**. Western blots, **B**. RT-PCR, **C**. Real-time PCR and **D**. ICC (100×) all showed that SULT1A1 was upregulated in T24 and EJ cells after resveratrol treatment. Ct, RV represented HBC cells treated without and with 100μM resveratrol incubation for 48h, respectively. * P<0.05, represents statistical significance between RV-treatment HBC cells and the normally cultured HBC cells, respectively.

Sulfation is an important metabolic pathway for xenobiotics and is catalyzed by the cytosolic sulfotransferases (SULTs). SULT1A1 appears to be an important phenol SULT because of its abundance and distribution in many tissues and wide substrate specificity [23, 24]. As shown in Figure 4, SULT1A1 expressed in normally cultured HBC T24 and EJ cells, and the densitometry scan of Western blots revealed that the SULT1A1 expression in RV-treated T24 cells (RV) increased about 1.7-fold and increased about 1.3-fold higher than that in RV-treated EJ cells (Figure 4A). The above results were further confirmed by PCR. RT-PCR showed the expression of SULT1A1 in RV-treated T24 and EJ cells was upregulated approximately 2-fold and 1.5-fold higher (Figure 4B), and was enhanced about 2.3-fold and 2.2-fold in real-time PCR (Figure 4C), respectively. ICC staining showed that SULT1A1 expressed in the cytoplasm of T24 and EJ cells, and the expression was both upregulated after 100μM RV treatment (Figure 4D).

### Decreased anticancer effects of RVS in HBC cells

**Figure 5:**
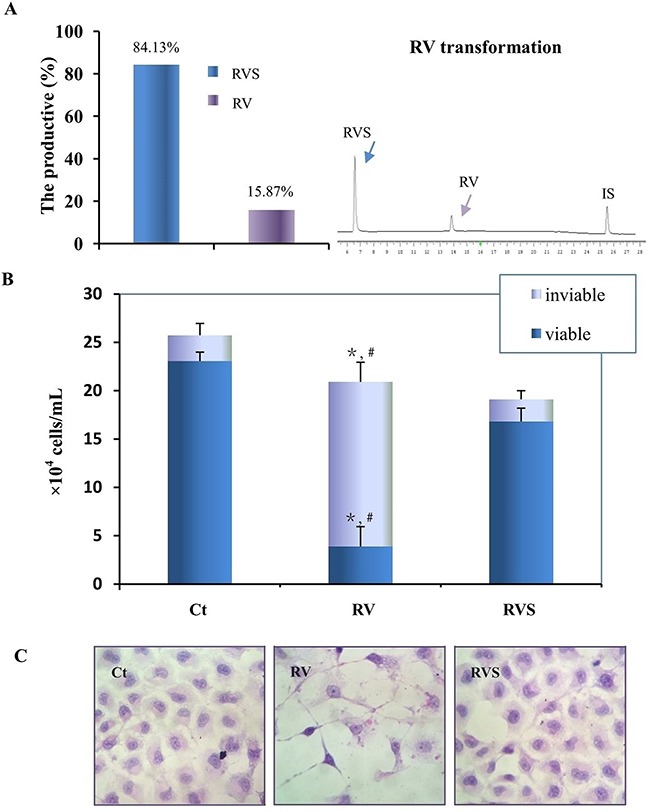
Biotransformation and bioactivity evaluation of RVS on T24 cells **A**. Quantification of the biotransformation efficiency of RVS (Left) by representative HPLC analysis (Right). RV, RVS and IS represent resveratrol, resveratrol monosulfate and 1,8-dihydroxy anthraquinone (Internal standard), respectively. **B**. Cells number was determined by Trypan Blue exclusion after normal culture (Ct), 100μM *trans*-resveratrol (RV), and 84.13% resveratrol monosulfate/15.87% *trans*-resveratrol mixture (RVS) incubation for 48h, respectively. The column indicates the number of viable cells. *, ^#^, RV treatment compared with Ct and RVS treatment, respectively (P<0.01). **C**. Morphologic evaluation of T24 cells incubated with normally culture (Ct), 100μM *trans*-resveratrol (RV), and resveratrol monosulfate/*trans*-resveratrol mixture (RVS) for 48h by HE staining (200×).

RVS was the main metabolite in T24 cells, and SULT1A1 distributed widely in many tissues. RVS was prepared with the homogenate of rat livers *in vitro* and identified by HPLC and LC/MS. The MS/MS analysis showed RVS was prepared successfully (Table 1), and HPLC analysis revealed that the RVS was the major component and about 84.13% of parent *trans*-resveratrol was biotransformed according to the chromatogram peak area (Figure 5A). T24 cells were treated with a final concentration of 100μM RVS mixture for 48h, but different from 100μM RV treatment, T24 cells showed neither distinct growth suppression nor the signs of cell apoptosis (Figure 5B, 5C).

**Table 1:** LC-MS/MS analysis of the compounds of resveratrol biotransformation

MS1 Ions (*m/z*)[M-H]^-^	MS2 Product Ions		Identification
	*m/z*	Fragment Loss	
227	185	[M-H-42]^-^	standard resveratrol
	143	[M-H-84]^-^	
227	185	[M-H-42]^-^	*trans*-resveratrol
	143	[M-H-84]^-^	
227	185	[M-H-42]^-^	*cis*-resveratrol
	143	[M-H-84]^-^	
307	227	[M-H-80]^-^	resveratrol monosulfate
	185	[M-H-80-42]^-^	
	143	[M-H-80-84]^-^	

### RV showed almost no side-effect to PBC cells

**Figure 6:**
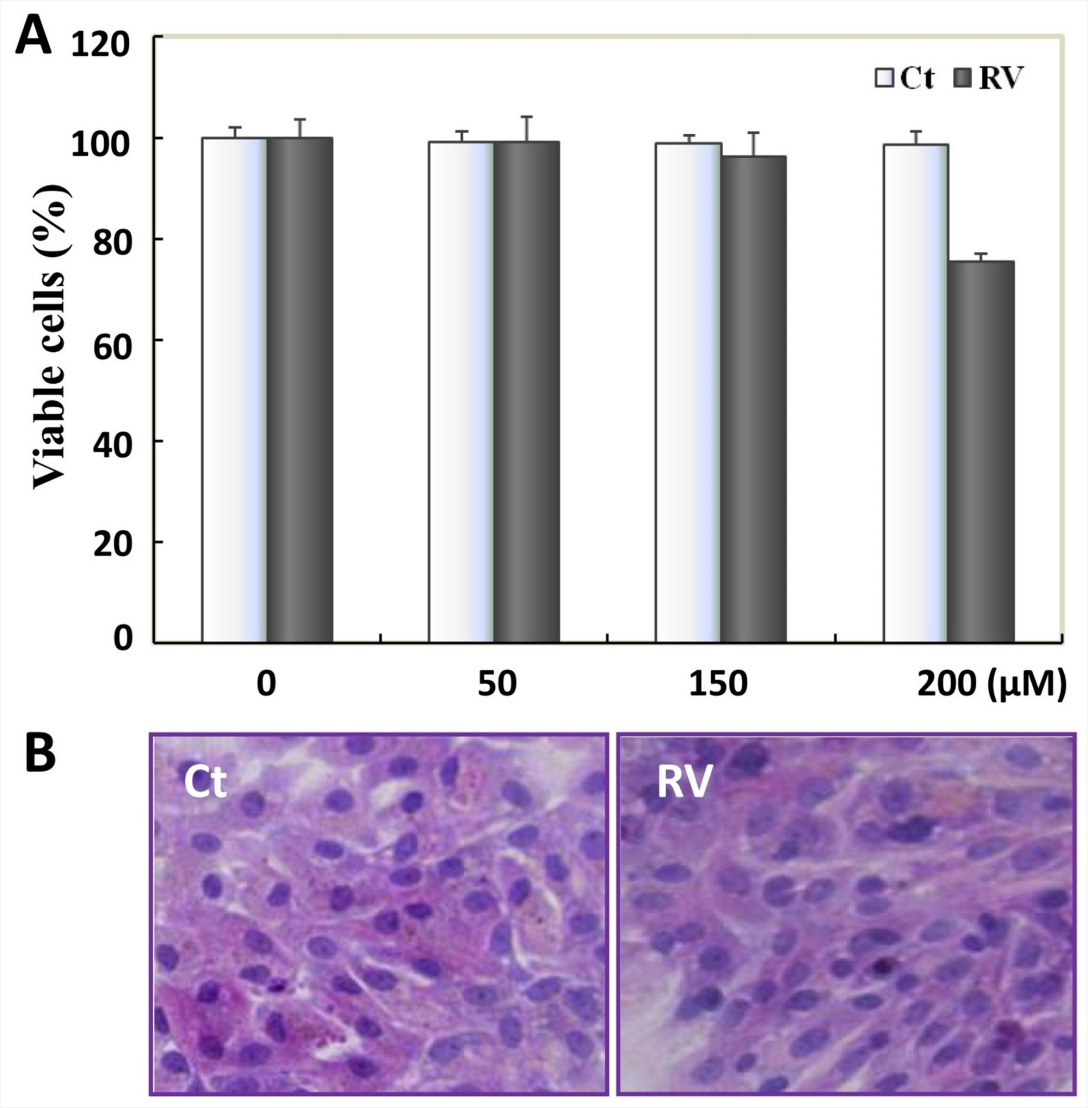
The safety evaluation of resveratrol to PBC cells **A**. Resveratrol's effect on the cell viability of the primary cultured normal rat bladder epithelial cells (PBC). Cells number was determined by MTT after 48h-100μM resveratrol incubation, data were expressed as means ± S.D. (n=3) (*, P<0.05). **B**. HE morphological staining performed on PBC cells with 100μM resveratrol treatment for 48h. PBC cells showed neither observable growth arrest nor morphological change. Ct, represented PBC cells were cultured in normal culture media; RV, represented PBC cells were treated with 100μM resveratrol treatment for 48h.

To explore whether RV has side-effect on primary cultured normal rat bladder epithelial cells (PBC), MTT and HE assay were carried out. The total number of PBC cells were about 250 000 (range: 250 000 cells/bladder to 350 000 cells/bladder) collected from twelve bladders. MTT results showed that the proliferation of PBC cells was not inhibited by 100μM-RV treatment, even tolerated as high concentration as 200μM-RV treatment (Figure 6A). After 100μM RV treatment for 48 hours, the condition media of the PBC cells were clear and the cells number increased. Compared with 48h-100μM RV treated T24 cells (Figure 5C), HE staining showed that PBC cells neither observable growth arrest nor morphological change after 48h-100μM RV incubation (Figure 6B), and the effective antitumor dosage 100μM RV displayed almost no side effect on PBC cells.

## DISCUSSION

Exploring RV's bioactive form has received more attention for “Resveratrol Paradox”, i.e., RV's low bioavailability but high pharmacological activity [9, 18]. So far, the confirmation trial of RV's metabolic active pattern *in vitro* and *in vivo* is still limited. Some researchers reported that piceatannol, which could be biotransformed from RV by cytochrome P450 CYP 1A1, 1A2 and 1B1 *in vitro* [25, 26], possessed more powerful biological activity [25, 27], but piceatannol as phase I metabolite was seldom detected in RV's metabolites [18, 28–30]. Though the vast majority of studies have been performed using RV parent compound, some researchers proposed that RV phase II metabolites might also possess the pharmacological activity for RV's low bioavailability [31, 32]. In the treatment of colon cancer cells, Aires *et al*. found RVS could inhibit colon cancer cells growth and accumulate cancer cells in S phase, but RV glucuronides (RV-3-O-glucuronide or RV-4’-O-glucuronide, Supplementary Figure 1) could not suppress cancer cells proliferation [31]. Almost at the same time, Polycarpou *et al*. evaluated the actions of RV and its metabolites on the growth of colon cancer cells *in vitro*. The results showed that RV could cause S phase arrest in all three cell lines (CCL-228, Caco-2 and HCT-116), RV 3-O-glucuronide and RV 4’-O-glucuronide caused G1 arrest in CCL-228 and Caco-2 cells, but RVS had no effect on cell cycle [32]. In addition to colon cancer, RVS also showed less bioactivity in human brain tumors (medulloblatoma UW228-3 and glioblastoma U251) and breast cancer cells (MB-MDA-231, ZR-75-1) [14, 17]. The above studies elucidated that RV parent compound could inhibit cancer cells growth, but whether RV sulfates or RV glucuronides playing the corresponding bioactivity would depend on the cancer cell types and the protocols used in the experiments.

RV showed the beneficial antitumor bioactivity in HBC cells [13, 33, 34], but how RV metabolized and whether RV or its metabolites (sulfates or glucuronides) exerted the corresponding effect on bladder carcinoma have not been reported so far. In this content, we found that HBC T24 and EJ cells showed different sensitivity to RV. Therefore, a comparison of RV metabolic patterns in RV sensitive and RV low sensitive carcinoma cells would be helpful to figure the issue out. *In virtue* of HPLC, LC-MS/MS and HRMS, we found RV was mainly metabolized into RVS in both HBC T24 and EJ cells (Figure 2), but it appeared that only a very small fraction of RV was metabolized to RVS after incubation with HBC cells, and the vast majority remained as the parent compound at 48h (Figure 3). Meanwhile, RVS was found as early as at 3h-time point both in T24 and EJ cells incubated with RV, but the T24 cells showed growth arrest until 24h-RV incubation and EJ cells still showed less morphological change (Figure 3, Supplementary Figure 2). According to the above results, the total amount of RV sulfate in cell lysate and supernatant didn't increase significantly compared with RV parent form, and the the metabolism was preceded to growth inhibition, which implied that the RV metabolites might not dominate significant anticancer bioactivity in HBC T24 cells.

RV was unstable, and its *cis*-form (*cis*-RV) was found both in the cell lysates and supernatant media of T24 cells (Figure 2A, Figure 3), but *cis*-RV did not exert bioactivity [17], so the operation was carried out under yellow light to avoid possible photochemical side-reactions. It is also known that resveratrol is a phytoestrogen, and phenol red in the culture media might show hormonal background, so we compared the cell culture of DMEM added with/without phenol red, to measure whether phenol red has interference effect on RV metabolism in HBC cells. As shown in Supplementary Figure 3, phenol red showed no interference impact on both RV metabolism and HBC cells growth, so we chose phenol red as indicator in this experiment.

For further evaluating the bioactivity of RV metabolites in HBC cells, the RVS was prepared by RV biotransformation *in vitro* [17, 35, 36], and was used to treat RV-sensitive T24 cells. Compared with the 100μM RV treatment, the T24 cells incubated with RVS mixture (84.13% RVS) did not show significant apoptotic characteristics and growth inhibition, thus, it indicated that RVS possessed less pharmaceutical effect on T24 cells. RV's pharmacological properties may result from activating or inhibiting the corresponding signaling pathways through cellular receptors [37]. It was reported that RV could inhibit the phosphorylation of Akt and decrease the expression of miR-21 in T24 and 5637 cells, thus induced HBC cells apoptosis via miR-21 regulation of the Akt/Bcl-2 signaling pathway [33]. Bai *et al*. also found RV could efficiently trigger HBC cells apoptosis through the modulation of Bcl-2 family proteins and activation of caspase 9 and caspase 3 followed by poly (ADP-ribose) polymerase degradation [21, 33]. In addition, the recent studies have demonstrated RV could activate SIRT1 *in vitro* by lowering its Michaelis constant (K_M_) [6, 38, 39], and more excited, Howitz *et al*. found RV could activate *in vivo* Sirt1 at very low concentration (nanomolar range) [38], which indicated RV could exert its multiple bioactivities though the low bioavailability. The above findings support our data that RV itself is more directly responsible for the antitumor activity, and RVS probably only be the metabolic form excreted from the HBC cells/tissues.

Drug metabolism is mainly classified into phase I and phase II reactions. So far, several polymorphic enzymes such as UDP-glucuronosyltransferases (UGTs), SULTs, N-acetyltransferases (NATs) are involved in the phase II metabolism of the xenobiotics in humans [40–42]. Since both T24 and EJ cells generate the same RV metabolite, and RVS is the major metabolite in HBC cells, which is catalyzed by SULTs, so the level of SULTs expression may positively or negatively influence RV's bioavailability. On the other hand, the role of genetic polymorphism on HBC risk has been investigated by several studies [43–46]. In 1999, Vineis *et al*. has reported that a possible association between metabolic polymorphisms and susceptibility to cancer [47]. Furthermore, similar results were found in the environmental exposures research, Hung *et al*. in 2004 investigated the effects of multiple genes including NAT, GST and SULT families, which showed clearly that the polymorphisms of these families may modulate individual response to bladder carcinogens and cancer susceptibility [48]. On the basis of overall limited evidence in human carcinogenicity data, the studies carried out that different population may be due to variability in the individual susceptibility to bladder carcinogens. Epidemiological and experimental evidence favors the important effects of gender, race and age on incidence and mortality of bladder cancer [49]. Particularly, a few surveys have investigated that several polymorphic enzymes are involved in the metabolism of the bladder carcinogens in humans. SULT1A1 is one of the families of genes encoding the polymorphic enzymes which are involved in the metabolism of bladder carcinogens in humans. It has been reported that the role of SULT1A1 in both the bioactivation and detoxification of various dietary and environmental mutagens may depend on the tissue or organ [50, 51]. It has been demonstrated that SULTs have substrate-dependent effects and they exhibit marked differences in tissue distribution as well as their sensitivity to thermal inactivation and inhibitor [51]. Moreover, previous studies have shown gender-specific differences for SULT activity [52–55]. Nowell *et al*. found that higher platelet phenol SULT activity in women than in men [56]. Additionally, in support of this, Klasaaen *et al*. observed a higher SULT1A1 mRNA expression in adult male rats than adult female rats [57]. Additionally, some studies provide epidemiologic evidence of a reduced bladder cancer risk in individuals with the SULT1A1 His^213^ allele genotypes which have been linked with an increased risk for cancer [58, 59]. All these studies elucidated that the gene variants which contribution in the inter-individual variations of genetic susceptibility to HBC could actually affect the metabolism of the relevant exposures in HBC. In this study, RV showed different sensitivity to the HBC T24 and EJ cells, comparing the expression levels of SULT1A1 which turned out to be closely but not directly related to the metabolic activity of RV, therefore, the different RV-responses of HBC T24 and EJ cells would be a possible association between metabolic polymorphisms and individual genetic susceptibility to cancer.

Since RV parent form was more directly responsible for RV's pharmacological activity, the safety profile of RV should be considered for further clinical application [13, 39, 60]. It was reported that RV was administered orally to male rats for 28d at a dose of 20mg/(kg X d), 1000 times the amount consumed by a 70kg person taking 1.4g of RV/d, but produced no adverse effect as assessed by growth, hematology, clinical chemistry, and histopathology [61]. In humans, a phase I study showed that ingestion of a single dose of RV (0.5g, 1g, 2.5g or 5g; 10 subjects per group) did not cause serious adverse events [61]. Recently, Anton SD and co-workers have conducted a double-blind, randomized, placebo-controlled trial to examine the safety in 32 overweight, older adults (mean age, 73±7 years). Compared with placebo, short-term (90 days) RV supplementation at doses of 300 mg/day and 1000mg/day does not adversely affect blood chemistries and is well tolerated in overweight, older individuals [62]. The above findings support the research of RV in larger clinical trials.

So far, the safety evaluation of RV on bladder has not been reported, therefore, the normal bladder epithelial cells were also treated with RV here, and the effective dosage of *trans*-RV (100μM) on bladder carcinoma cells showed no adverse effect on normal bladder cells. Especially, the bladder is a well-defined cavity organ in the anatomical location, so regional intravesical instillation is highly conducive to HBC therapy. At diagnosis, nearly 80% of bladder carcinomas are superficial and usually treated with drug adjuvant intravesical therapies after cystectomy to delay or prevent recurrence [63]. The good lipophilicity of RV makes it easy to be well absorbed by bladder endothelial cells via simple diffusion, and treatment with the effective doses of RV was well tolerated by the PBC cells, therefore, in view of the excellent safety of RV [61, 62, 64–66], intravesical instillation of RV would provide the potential clinical value for bladder carcinoma prevention and treatment.

## CONCLUSIONS

In conclusion, HBC T24 cells showed higher sensitivity to RV than EJ cells, but both of them produced the same metabolite, RVS. RV parent form, rather than its metabolite (RVS), was primarily responsible for the anti-bladder carcinoma activity. RV's associated metabolic enzyme SULT1A1 was upregulated after RV treatment in different sensitive HBC cells, and that indicated that neither RV's metabolic pattern nor the related metabolic enzyme SULT1A1 was correlated with the different RV sensitivities of HBC T24 and EJ cells. In addition, RV showed almost no side-effect on the rat normal bladder epithelial cells at the therapeutic dosage, so compared to RV metabolites, *trans*-RV would be a potential pharmacological medicine in bladder cancer clinical prevention and therapy. In consideration of variable responses of T24 and EJ cells to RV, therefore, individual cancer types should be regarded as an important factor in medicine application. Meanwhile, in view of RV's bioavailability was affected by different administration routes [67], the appropriate administration routes and drug delivery carriers should be focused on to improve RV's bioavailability.

## MATERIALS AND METHODS

### Cell culture and treatment

HBC T24 and EJ (human bladder transitional cell carcinoma) were purchased from the Type Culture Collection of the Chinese Academy of Sciences (Shanghai, China)/American Type Collection (ATCC, Rockville, USA). Cells were cultured in high glucose Dulbecco's modified Eagles medium (DMEM; Invitrogen Co., Grand Island, NY, USA) which were supplemented with 10% fetal bovine serum (Gibco Life Science, Grand Island, NY, USA) and 1% penicillin-streptomycin (Gibco Invitrogen Corp., Grand Island, NY, USA) under standard conditions at 37°C in a humidified atmosphere containing 5% CO_2_ and 95% air. The cells were plated in 100 mm dishes (Nunc A/S, Roskilde, Denmark) at the density of 50,000/ml and incubated for 24h before further experiments. For morphologic evaluation and immunocytochemistry (ICC) staining, the coverslips were put into the dishes before initial cell seeding and collected after treatment with/without RV (Sigma Chem Co., St. Louis, MO, USA) in the experiments.

To efficiently dissolve RV, dimethyl sulfoxide (DMSO; Sigma Chem Co., St. Louis, MO, USA) was chosen as a solvent. For *trans*-RV was sensitive to natural light and ultraviolet light [16], a stock solution of 100mM *trans*-RV was wrapped in aluminum foil for protection against light and stored at -20°C. It would be diluted with culture media to the optimum working concentrations just before use. 0.2% DMSO was used to incubate with the cells as the background control, which caused no measurable effect on cell growth.

### Primary urinary bladder transitional cell culture

The healthy Wistar rats were obtained from the Experimental Animals Center of Dalian Medical University, which were fed in cages under controlled conditions maintained at 22°C with a 12h light/dark period. All experimental protocols had been reviewed and approved by the ethics committee of Dalian Medical University for the protection of human subjects and experimental animals before conducting the project. The rats were sacrificed using CO_2_ asphyxia, and the bladders were freshly removed and treated with trypsin and ethylene diamine tetraacetic acid (EDTA), then the primary cultured normal rat bladder epithelial cells (PBC) were selectively harvested for the further experiments [68]. The cells were cultured in high glucose DMEM supplemented with 10% fetal bovine serum and 1% penicillin-streptomycin, under standard conditions at 37°C in a humidified atmosphere containing 5% CO_2_ and 95% air.

### Sensitivity evaluation of RV

Cell viability were determined by 3-(4,5-dimethy- lthiazol-2-yl)-2,5-diphenyltetrazolium bromide (MTT; Sigma-Aldrich Co., USA) assay [69]. HBC T24 and EJ cells were seeded in 96-well plates with DMEM medium supplemented 10% fetal bovine serum. After overnight incubation at 37°C, the cells were treated with various concentrations of RV (0-200μM), and 0.2% DMSO was used as background control. After incubation with RV or DMSO for 6h, 12h, 24h and 48h, respectively, the cell absorbance data were measured by a spectrophotometer (Thermo Fisher Scientific, USA) at 490nm. After being treated with 100μM RV for 48h, the cells were collected for flow cytometry (FCM) analysis [70]. Meanwhile, cell-bearing coverslips were harvested and fixed properly for hematoxylin and eosin (HE) and ICC staining. To establish the confidential conclusions, each of the experimental groups was set in triplicate and the experiments were repeated at least three times.

### Sample preparation and HPLC analysis

After treatment with 100μM RV, the cell-free culture media and the cells were harvested, respectively. The cells were washed three times with PBS (phosphate buffered saline solution, pH 7.4) and lysed by sonication [71]. Then the cell lysates and their condition media were centrifuged at 10,000g for 5min, and followed by SPE [72]. In brief, the samples were loaded onto the Cleanert PEP-SPE cartridges (60mg; Agela Technol Inc. PA, USA), which were previously activated with methanol (Fisher Sci, Fair Lawn, NJ, USA) and ultrapure water purified with a Milli-Q water purification system (Millipore, Bedford, MA, USA), then the cartridges were subsequently washed with ultrapure water. The samples absorbed in the cartridge were eluted with methanol, and the eluate was dried by nitrogen spraying. At last, the residues were dissolved in 200μl of methanol for HPLC and LC-MS analysis. In all cases, sample manipulation was performed in the dark to minimize the possible photochemical isomerization of *trans*-RV to its *cis*-form [17, 73].

Altogether four kinds of samples were subjected to HPLC analysis: Sample 1, RV standards; Sample 2, RV-containing media as background control; Sample 3, the culture media incubated with T24 or EJ cells after 100μM RV treatment for 48h; Sample 4, the T24 or EJ cells treated with 100μM RV for 48h. The determination of the samples was performed on the Agilent 1200 HPLC system (Agilent Technologies, Santa Clara, CA, USA) consisted of an Agilent 1260 binary pump and 1260 dual wavelength UV-Vis detector. The detection was carried out at a wavelength 303nm and the column oven was set at 30°C [64]. Chromatographic separation of the samples was performed on a Cosmosil C18-AR-II column (5μm, 4.6mm×250mm; Nacalai Tesque, Japan) preceded by a C18 guard column (5μm, 4.6mm×10mm), with a mobile phase consisted of 5mM ammonium acetate (mobile phase A, Alfa Aesar, A Johnson Matthey Company, Ward Hill, MA, USA) and methanol (mobile phase B) at a flow rate of 1ml/min. The mobile phases were degassed by sonication for 15min at room temperature before use. A gradient elution was carried out as follows: 0min, 45% B; 25min, 60% B; 30min, 45% B; 40min, 45% B. Subsequently, equilibrate the column for 10min before the next injection. Samples were filtered with a 0.45μm filter membrane (Millipore, Bedford, MA, USA) and a 10μl aliquot was injected.

For quantitative analysis, cells were scraped off, washed three times with PBS (pH 7.4), and lysed with 416μl PBS and 84μl IS (1, 8-dihydroxyanthraquinone, 200μg/ml) by sonication. Cells cultured for 48h in medium with the same working concentration of DMSO (0.2%) were used as a background control. The collected culture medium and cell lysates were centrifuged at 12, 000 rpm/min for 10 min at 4°C. The supernatant was collected, and then purified with SPE. The eluates were evaporated to a final volume of 400μl for HPLC analysis. Chromatographic condition: The analyses were performed on the HITACHI Chromaster 5000 HPLC system (Hitachi High-Technologies Corporation, Tokyo, Japan) consisted of a HITACHI 5110 pump, 5210 auto sampler and 5430 diode array detector. The detection was carried out at a wavelength 303nm and 5310 column oven was set at 30°C. All the separation of the samples was carried out on a Cosmosil C18-AR-II column (5μm, 4.6mm×250mm; Nacalai Tesque, Japan) with a mobile phase consisted of 20% acetonitrile (mobile phase A, acetic acid adjusted pH 3.5) and 80% acetonitrile (mobile phase B, acetic acid adjusted pH 3.5) at a flow rate of 1ml/min. The mobile phase consisted of two phases, phases A was 20% acetonitrile and phase B was 80% acetonitrile (acetic acid adjusted pH 3.5). The gradient elution mode was carried out as follows: 0-14 min, linear gradient from A: B (0: 100, v/v) to A: B (60: 40, v/v); 14-20 min, the liner gradient from A: B (60:40, v/v) to A: B (0: 100, v/v), the mobile phase was hold on A: B (0: 100, v/v). Each run was followed by equilibration time of 15min before the next injection [67].

### Identification of RV metabolite(s) by LC-MS/MS and HRMS

To further identify the metabolite(s) of RV in T24 cells, the extracted samples were analyzed by direct online LC-MS/MS under the chromatographic series (Agilent Technol Inc., Santa Clara, CA, USA) coupled to an Applied Biosystems API 3200 QTrap tandem mass spectrometer (Applied Biosystem/MDS SCIEX, Foster City, CA, USA). The MS and MS/MS data were obtained by the Applied Biosystem/MDS SCIEX analyst software (Version 1.4.1). A Cosmosil C18-AR-II column (5μm, 4.6mm×250mm; Nacalai Tesque, Japan) with a guard column was used for chromatographic separation. 5mM ammonium acetate was used as solvent A, and methanol as solvent B with the following gradient at a flow rate of 500μl/min: 45-60% B linear (0-25min), 60-45% B linear (25-30min), 45% B linear (30-40min).

The MS determination of the metabolites was operated in a negative ion mode. To obtain maximum sensitivity, the ion spray interface and the mass spectrometric parameters were optimized before use. Full-scan data acquisition was performed by scanning over the range of m/z 100-600 in profile mode, using a cycle time of 2s and a pause between scans of 2ms [17]. The identification of the samples was based on their retention time and ion fragmentations in the MS and MS/MS mode.

For further confirmation of the metabolites, HRMS analysis was performed on the LC-ESI-IT-TOF-MS (Shimadzu Co., Kyoto, Japan) in negative ion mode at a resolution of 10,000 FWHM. Before the sample was injected onto a Shim-pack VP-ODS column (5μm, 2.0×150 mm; Shimadzu Co., Kyoto, Japan), the accurate masses were corrected by using the standard sample sodium trifluoroacetate. The column temperature was kept at 40°C, and the flow rate was 0.6ml/min. The mobile phase consisted of two phases: phase A was acetonitrile/10mM acetic acid water solution (95:5, v/v, pH 3.0), and phase B was 10mM acetic acid water solution (pH 3.0). A gradient elution was carried out as the following proportions (v/v) of phase A and B: 0min (95/5), 5min (70/30), 5.5min (40/60), 12.5(40/60), 12.6min (95/5). The column was equilibrated with 5% phase B for 5min before the next run. MS data were processed with LC-MS solution ver. 3.4 software (Shimadzu, Japan).

### Bladder-associated metabolic enzyme determination by ICC and western blots

The main metabolite of RV in HBC T24 and EJ cells was RVS, since sulfation was regulated by phase II metabolic enzyme sulfotransferases (SULTs), and SULT1A1 took part in phenol compounds metabolism [23, 24], thus evaluating the potential influence of RV to SULT1A1 appeared to be essential. To evaluate RV's related metabolic enzyme SULT1A1 in T24 and EJ cells, ICC staining and Western blots were performed. For ICC staining, cells on coverslips were collected from different experimental groups treated with/without RV. The first antibody of rabbit anti-human SULT1A1 (Protein Tech Group, Inc., Chicago, USA) was used in the dilution rates of 1:120. Other solutions were prepared by ICC staining kit (HistotainTM-plus Kits SP-9000, Zymed, USA).

For Western blots analysis, total cellular proteins were prepared from the treated cells. 50μg of the sample proteins were separated by electrophoresis in 10% sodium dodecylsulfate-polyacrylamide gel electrophoresis (SDS-PAGE) and transferred to polyvinylidene difluoride membrane (Amersham Biosci, Buckinghamshire, UK). The membrane was blocked with 5% skimmed milk in TBS-T (10mM Tris-Cl, PH 8.0, 150mM NaCl and 0.5% Tween 20) at 37°C for 2h, followed by incubation with the first antibodies in the appropriate concentrations (SULT1A1, 1:1000; β-actin, 1:3000, Protein Tech Group, Inc., Chicago, USA) at 4°C overnight, and then incubated with HRP-conjugated anti-rabbit IgG (Zymed Lab Inc., San Francisco, CA, USA) at 37°C for 1.5h. The immunolabeling was detected using the enhanced chemiluminescence system (Roche Diagnostics GmbH, Mannheim, Germany) and visualized using the UVP Bio-spectrum Imaging System (UVP, Inc, Upland, CA, USA). *β*-Actin was used as an internal quantitative control in densitometry analysis. After removing the labeling signal by incubation with stripping buffer (62.5mM Tris-Cl, pH 6.7, 100mM 2-mercaptoethanol, 2% SDS) at 55°C for 30min, the membrane was re-probed with *β*-actin by the same experimental procedures until all of the parameters were examined.

### RT-PCR and real-time PCR

For semi-quantitative determination of SULT1A1 mRNA expression, total cellular RNA was extracted from cells with Trizol reagent (Life Technologies, Grand Island, NY, USA). Reverse transcription was performed on RNA samples, and this was followed by PCR for SULT1A1 and *β*-actin, according to the manufacturer's recommendations (Takara, Dalian Branch, Dalian, China). The sequences of PCR primers were as follows: SULT1A1, Forward: 5’-GCAACGCAAAGGATGTGGCA-3’, Reverse: 5’-TCC GTAGGACACTTCTCCGA-3’. *β*-actin, Forward: 5’-GC ATGGAGTCCTGTGGCAT-3’, Reverse: 5’-CATGAAG CATTTGCGGTGG-3’ [17]. The PCR products were resolved on 1% agarose gel containing ethidium bromide (0.5μg/ml), and photographed with the UVP Bio-spectrum Imaging System. The PCR products generated from the same reverse transcription solutions by a pair of *β*-actin primers were used as internal quantitative controls.

For quantitative real-time PCR, RNA samples (1μg) were reversely transcribed in a final volume of 20μl containing Prime Script RT reagents (Takara, Dalian Branch, Dalian, China). Reaction mixtures were then incubated at 37°C for 15min and 85°C for 5s, and kept at 4°C. The primers were as follows: SULT1A1, Forward: 5’-GCAACGCAAAGGATGTGGC-3’, Reverse: 5’-TC CCTTTTCGGGTTCTCCTTC-3’. GAPDH, Forward: 5’-GAAGGTCGGAGTCAACGGAT-3’, Reverse: 5’-CC TGGAAGATGGTGATGGG-3’. 25μl reaction mixtures were prepared by adding 2×SYBR Premix Ex TaqTM II (Takara, Dalian Branch, Dalian, China), 10μM forward and reverse primers (Takara, Dalian Branch, Dalian, China), 2μl of cDNA template, and a suitable amount of distilled H_2_O. Amplification and detection were performed with the Thermal Cycler Dice Real Time System (TaKaRa; Code TP800). Each reaction was performed in triplicate, and ‘no-template’ controls were included in each experiment.

### RV biotransformation and anticancer evaluation

For sulfonating RV, the homogenate of rat livers with ice-buffer (250mM sucrose, 10mM HEPES, 3mM 2-mercaptoethanol, pH7.4) was centrifuged at 10,000×g for 20min in 4°C, followed by supernatant was centrifuged at 100,000×g for 60min in 4°C to get cytosolic proteins which stored in -80°C until use (less than 6 months) [35]. Subsequently, a mixture (200μl) containing 100μl cytosolic proteins isolated from rat livers, 5mM RV, 2mM 3’-phosphoadenosine 5’-phosphosulfate (PAPS, Sigma Chem Co., St. Louis, MO, USA), 1mM dithiothreitol (DTT) and 20mM Mops buffer was prepared and incubated at 37°C for 2h. The reaction was terminated by standing the mixture-containing thin-wall tube in the boiling water for 5min. After the suspension was centrifuged at 10,000×g for 5min, 10μl of the supernatant was subjected to HPLC and LC/MS analysis for separating RV and its biotransformation metabolite(s), and the aliquots of the remaining part was used to treat T24 cells in the total concentration of 100μM. The cells treated with the chemical solution for liver lysate preparation, the liver lysate alone and the combination of RV were used as background controls, respectively. The cellular response(s) were checked with the parameters mentioned above.

### Statistical analyses

Data were given as the mean ± standard deviation, and statistical analyses were performed using SPSS 13.0 and GraphPad Prism 5. MTT data were analyzed with one-way ANOVA. It was considered statistically significant if the *p*-value is less than 0.05.

## SUPPLEMENTARY MATERIALS FIGURES AND TABLES


