# Supplementary material for: Differential sensitivities of bladder cancer cell lines to resveratol are unrelated to its metabolic profile
Source: Oncotarget. 2017 Feb 2;8(25):40289–304. doi: 10.18632/oncotarget.15041 (PMC5522211; doi:10.18632/oncotarget.15041)
Supplement: Supplementary file 1 [file oncotarget-08-40289-s001.pdf]

Differential sensitivities of bladder cancer cell lines to resveratol are unrelated to its metabolic profile

SUPPLEMENTARY MATERIALS

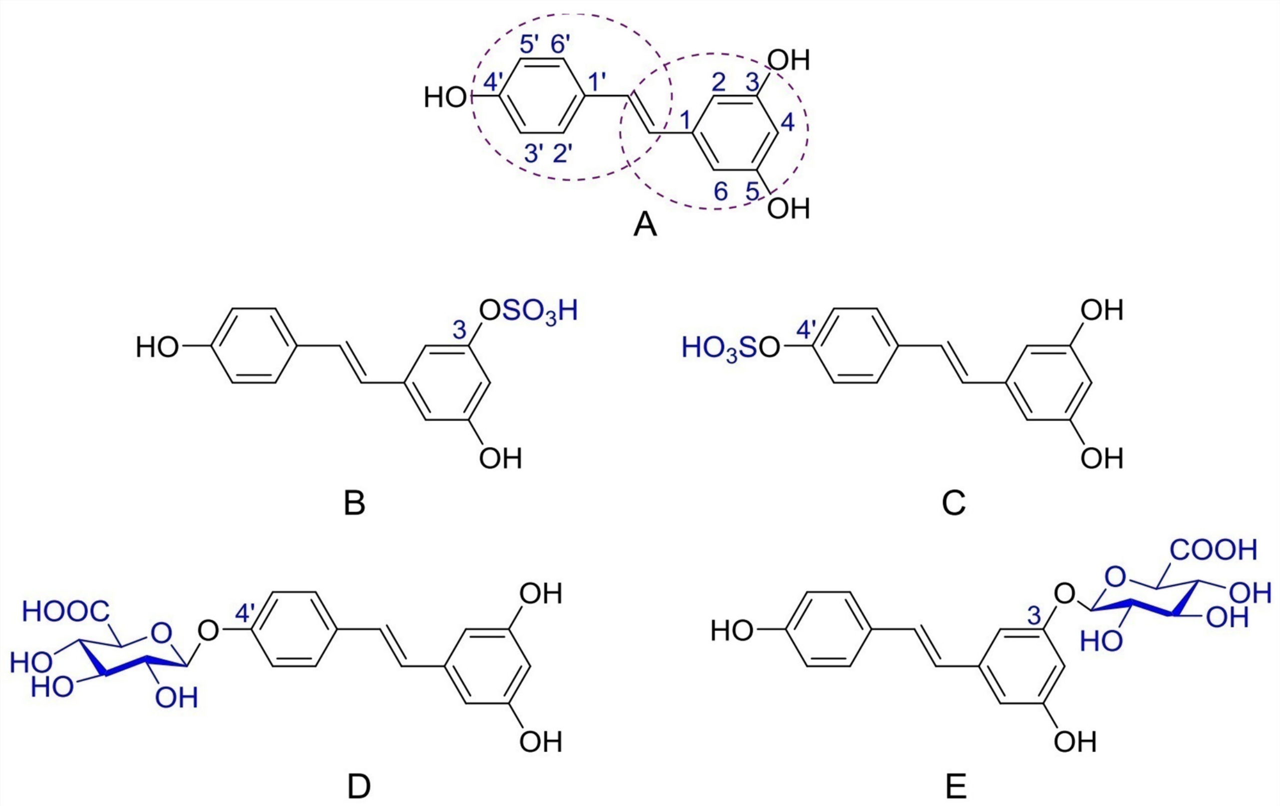

Supplementary Figure 1: Chemical structures of resveratrol and its metabolites. A. *trans*-resveratrol; **B.** *trans*-resveratrol-3-O-monosulfate; **C.** *trans*-resveratrol-4’-O-monosulfate; **D.** *trans*-resveratrol-4’-O-monoglucuronide; **E.** *trans*-resveratrol-3-O-monoglucuronide.

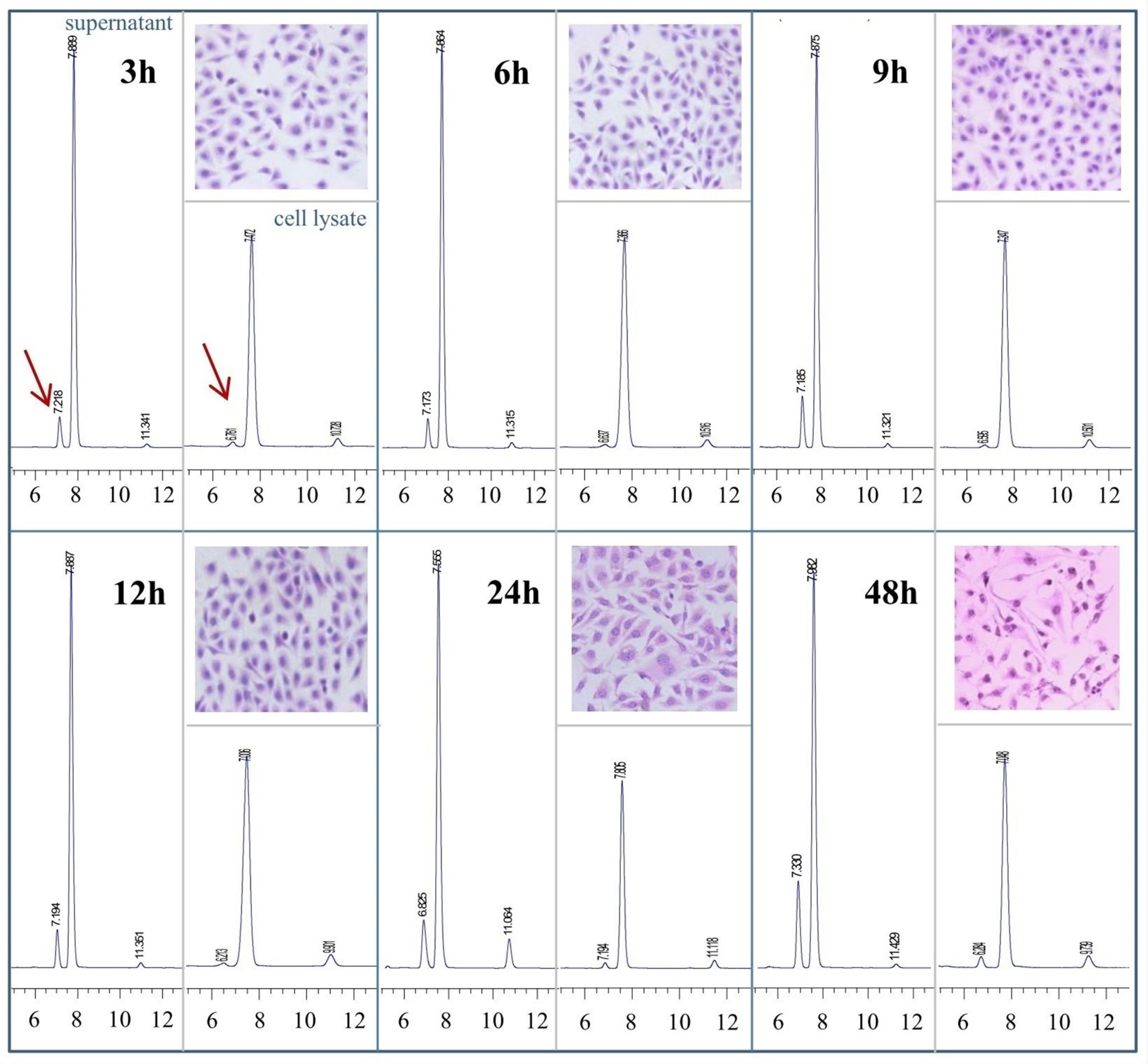

Supplementary Figure 2: Resveratrol metabolic process in EJ cells. Resveratrol metabolites in EJ cells were analyzed by HPLC after 100μM resveratrol treatment for 3, 6, 9, 12, 24 and 48h, respectively. RVS could be detected as early as 3h of drug treatment both in supernatant and cell lysates. Morphologic change was evaluated by HE staining (100×), and EJ cells showed neither observable growth arrest nor morphological change during 48h resveratrol incubation.

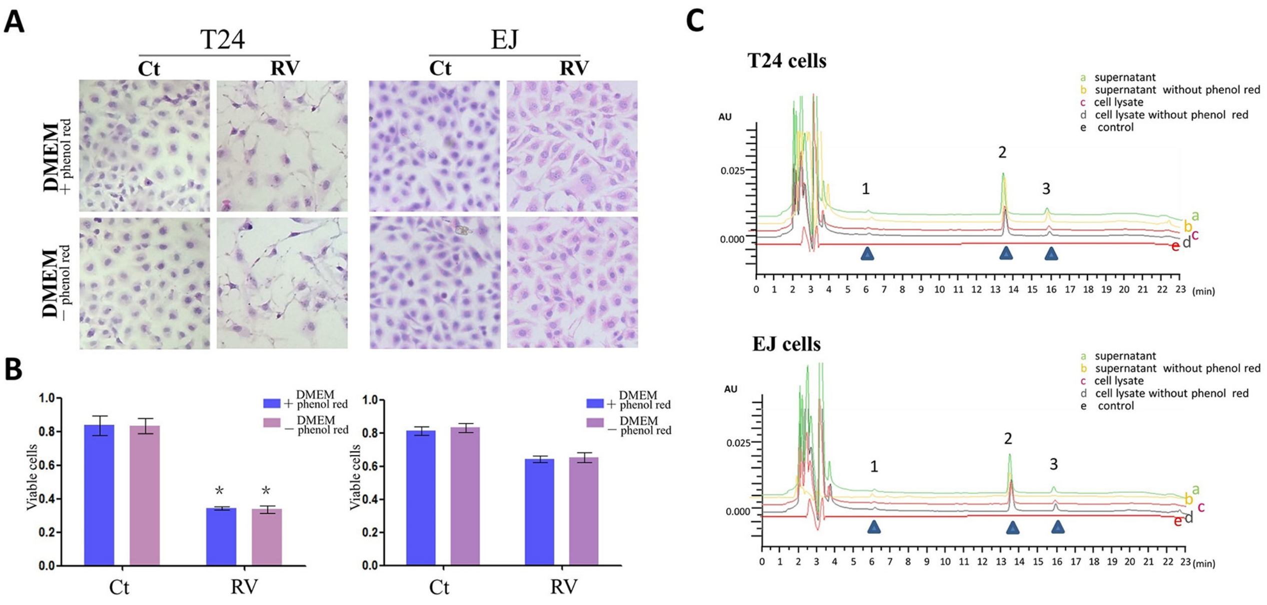

Supplementary Figure 3: Phenol red showed no effect on RV-treatment T24 and EJ cells. Studies have been reported that resveratrol has estrogenic activity and phenol red in DMEM has the same effect as resveratrol does, therefore, (-) phenol red DMEM was used to rule out the possibility of interaction interference between anti-carcinoma effects of resveratrol and estrogenic activity of phenol red. **A.** The effects of resveratrol morphological changes of T24 and EJ cells by HE morphological staining. Cells at a density of 4×10^5 cells per well were placed in dishes with coverslips, then T24 and EJ cells were treated without (N)/with (R) 100µM resveratrol and with/without phenol red DMEM for 48h. Cells coverslips were harvested for examination and no obviously change observed between with/without phenol red DMEM both in T24 and EJ cells. **B.** Effect of resveratrol treatment on HBC T24 and EJ cells. Cells were incubated with 100µM resveratrol and treated with/without phenol red DMEM, respectively, and then cells number was determined by MTT as described in the Materials and Methods. Data were presented as mean±S.D. of three independent experiments. Bars means Standard Errors, *P<0.05 reveal significant different compared with the control. **C.** HPLC chromatography analysis. (a) The supernatant of T24 or EJ cells was analyzed after incubation with 100µM resveratrol for 48h (M1, t_R=6.257; M2, t_R=13.647; M3, t_R=16.160). (b) The supernatant without phenol red of T24 or EJ cells was analyzed after incubation with 100µM resveratrol for 48h; (c) The T24 cells lysate was analyzed after incubation with 100µM resveratrol for 48h; (d) The T24 cells lysate was analyzed after incubation with 100µM resveratrol and DMEM without phenol red for 48h; (e) The blank culture media was set as control.

Supplementary Table 1: Resveratrol metabolite analysis in HBC T24 and EJ cells

Metabolites	T24		EJ	
	Cell lysates (%)	Supernatant (%)	Cell lysates (%)	Supernatant (%)
Trans-resveratrol	94.222±0.320	87. 828±0.669	92.415±0.690	82.172±0.789
Cis-resveratrol	4.738±0.284	4.104±0.179	4.991±0.272	6.686±0.172
Resveratrol monosulfate	1.040±0.328	8.068±0.785	2.594±0.948	11.142±0.950
